# The impact of health literacy on medico‐social follow‐up visits among French cancer survivors 5 years after diagnosis: The national VICAN survey

**DOI:** 10.1002/cam4.3074

**Published:** 2020-04-23

**Authors:** Youssoufa M. Ousseine, Anne‐Déborah Bouhnik, Patrick Peretti‐Watel, Aline Sarradon‐Eck, Victoria Memoli, Marc‐Karim Bendiane, Marie‐Anne Durand, Julien Mancini

**Affiliations:** ^1^ Aix Marseille Univ, INSERM, IRD, SESSTIM, Sciences Economiques & Sociales de la Santé & Traitement de l’Information Médicale Marseille France; ^2^ INSERM UMR VITROME Aix‐Marseille University SSA IRD Marseille France; ^3^ Institut Paoli‐Calmettes UMR1252 SESSTIM CANBIOS Marseille France; ^4^ The Dartmouth Institute for Health Policy and Clinical Practice Dartmouth College Hanover NH USA; ^5^ APHM, Hop Timone BIOSTIC Marseille France

**Keywords:** cancer survivors, follow‐up, health literacy, medico‐social, VICAN survey

## Abstract

**Background:**

Long‐term medico‐social follow‐up of cancer survivors is a challenge because of frequent subsequent troubles. In particular survivors with lower health literacy (HL) have poorer health and might more often use primary care services. However, the impact of HL on cancer survivors’ medico‐social follow‐up visits is not known. Our aim was to study medico‐social follow‐up and its associated determinants with a focus on HL 5 years after diagnosis.

**Methods:**

VICAN is a national survey of French adult cancer survivors 5 years after a primary cancer. The Single‐Item Literacy Screener was used to define functional HL in this sample. We also asked patients to report the frequency of follow‐up visits with a general practitioner (GP) and/or social worker (SW) regarding their cancer disease.

**Results:**

The 4045 participants were 57.4 ± 12.9 years old at diagnosis (range 20‐82) and 1495 (37%) were classified as having inadequate HL. Most cancer survivors (66.7%) were followed up by a GP regarding their cancer while only 14.5% had contact with a SW. After adjustment for sociodemographic, medical, and psychosocial characteristics, medico‐social follow‐ups (GP and SW visits) were more frequent among survivors with low HL. Furthermore, low income, unemployment, impaired mental health, treatment by chemotherapy, and perception of sequelae and fatigue were also associated with more frequent medico‐social follow‐up. Cancer localization association with medico‐social follow‐up was heterogeneous.

**Conclusion:**

French cancer survivors with limited HL, lower socioeconomic status, and more severe cancer were more likely to use GP care and social services. Raising awareness and training GPs and SWs on medico‐social follow‐up for patients with limited HL seem necessary to support these vulnerable survivors.

## Graphical abstract

Among French cancer survivors, 37% had a limited health literacy level. They were more likely to use GP care and social services. Raising awareness and training general practitioners and social workers on medico‐social follow‐up for patients with limited health literacy seem necessary to support these vulnerable survivors.

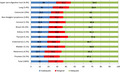

## 1 INTRODUCTION

The prevalence of cancer is rising globally.^1^ As cancer has often evolved from a life‐threatening disease into a chronic illness, a large number of patients will therefore become long‐term survivors. However, cancer diagnosis and treatments affect patients’ life. Cancer survivors often suffer from anxiety, depression, as well as other sequelae and side effects of cancer and its treatment.^2^, ^3^ Psychosocial and financial issues are often overlooked due to the inability to work during and after treatments.^4^

Long‐term follow‐up of cancer survivors is becoming a challenge.^5^ In countries like France, where the general practitioner (GP) is at the center of primary care, the medical authorities suggest integrating the GP very early in the oncology care of their patients so that later these GPs continue to ensure the long‐term follow‐up of cancer survivors.^6^ Several studies suggested transferring long‐term follow‐up of cancer survivors to primary care, with a clear survivorship care plan.^5^, ^7^ Furthermore, financial and psychosocial difficulties require support by a social worker.^8^, ^9^ The GP and SW can play a central role in the care of cancer survivors. In France, access to GP and oncologist is free. In public hospitals and specialized care centers, access to a specialist is completely free of charge but the delay to get an appointment can be long. To overcome these difficulties, some patients use specialists in the private sector. However, access to specialists in private sector may result in an advance of fees pending reimbursement or in out‐of‐pocket costs for the rare patients who does not have complementary (universal or private) health insurance.^10^

Access to a SW is not systematic. Some cancer centers may offer the patient social support and others may not. The patient may also seek the assistance of a SW if he/she deems it necessary. In recent years, in a multidisciplinary management framework, clinics have begun to systematize the integration of SWs and GPs into the management of their cancer patients.

However, there is little work evaluating the long‐term reintegration of cancer survivors into the primary care system, social support, and the associated determinants.^11^, ^12^, ^13^, ^14^ These studies showed an increased utilization of primary care, particularly the GP visit of cancer survivors. Primary health‐care use in cancer survivors was particularly high for younger patients and those without chronic disease,^14^ and mostly related to common infections and acute symptoms, which may be due to direct effects of cancer treatment or increased health concerns.^13^ A better use of health care requires that patients have an adequate level of health literacy (HL).^15^ Research also suggests that limited HL is globally associated with poorer health ^16^, ^17^and increased health service utilization.^18^ The difficulties of low literate patients to understand health information, to navigate the health‐care system, ^19^, ^20^, ^21^, ^22^, ^23^ and sometimes to adhere to recommended treatments require a specific support that may be provided by primary care and social workers. Indeed, we would expect more medico‐social follow‐up visits among cancer survivors with limited HL.

Our aim was to study medico‐social follow‐up and its associated determinants with a focus on HL among cancer survivors, using a national representative sample of cancer survivors in France 5 years after diagnosis.

## 2 MATERIAL AND METHODS

### 2.1 Data source

VICAN is a national survey of French cancer survivors, aged 18‐82, diagnosed with primary cancer between January and June 2010, registered in the Long Duration Disease File of the French National Health Insurance (FNHI) File (ALD file), and belonging to one of the three main French Health Insurance systems, which cover >90% of the population. The ALD file managed by the FNHI provides an almost exhaustive list of individuals diagnosed with cancer, since it allows insured persons suffering from cancer to benefit from 100% coverage of all cancer‐related costs by the FNHI.

VICAN survey was restricted to 12 cancers (prostate, melanoma, bladder, endometrial, thyroid, kidney, breast, cervical, non‐Hodgkin lymphoma, colorectal, lung, and upper aero‐digestive tract) accounting for 88% of cancer incidence in France.^24^ The data analyzed here were collected from survivors’ interviews 5 years after diagnosis. Participants were interviewed using a computer‐assisted telephone interview (CATI) system. A postal questionnaire was proposed to people with lung or upper aero‐digestive tract cancer, as their condition could have hampered their ability to respond orally. The methodology and details of the VICAN survey have been extensively described in a previous article.^24^ VICAN survey data are available through French National Cancer Institute (INCa).

### 2.2 Weighting procedure

A weighting procedure was applied to ensure representativeness at a national level and to minimize some nonresponse bias. First, to ensure representativeness of the target population with respect to age, health insurance regime, and cancer site, weights were calculated as the inverse of the sampling rates using national health insurance information system.^25^ Then, an adjustment of these weights was made to minimize some nonresponse bias by comparing the characteristics available for both respondents and non‐respondents. The following elements have been taken into account: health insurance regime, age, gender, cancer site, severity of the disease at the time of the survey, and the socioeconomic deprivation index.^24^

### 2.3 Measures

#### 2.3.1 Dependent variables

##### Medico‐social follow‐up

The medical follow‐up corresponded to follow‐up visits by a GP and was evaluated with a question asking, “*Do you consult your general practitioner for the follow‐up of your cancer?*” Social care follow‐up was also self‐reported asking, “*Following your cancer, have you been in contact with a social worker?*”

#### 2.3.2 Independent variables

##### Health literacy

The health literacy level was evaluated using the Single Item Literacy Screener (SILS).^26^ Response categories were merged to end with three categories: adequate, marginal, and inadequate HL.

##### Sociodemographic characteristics

Age at diagnosis, gender, marital status, education level, employment status at time of the survey, income, and simplified socioeconomic deprivation index. This index is constructed from patients’ living area indicators that reflect the multiple aspects of socioeconomic status (income, employment, housing, family and household, and educational level) in order to discriminate between socially vulnerable neighborhoods from more advantaged ones.^27^ This index ranging between 0 (low deprivation areas) and 1 (high deprivation areas) allowed us to categorize survivors into low (<tertile 1), medium (tertile 1‐2), or high (>tertile 2) socioeconomic deprivation groups. Furthermore, we measured the level of perceived precariousness (financial comfort) of participants through a question asking, *‘Would you say that financially…’ (‘you are comfortable’; ‘it's okay’; ‘you have to be careful’; ‘you can hardly do it’;* and *‘you can't do it without incurring debt’)*.

##### Clinical characteristics

Initial treatments, cancer locations, and the presence of metastasis at diagnosis were collected. Self‐reported sequelae were estimated using a question asking: *In general, do you have any sequelae following the care of your illness*? Possible responses were as follows: ‘*don't know’; ‘yes and they are very important’, ‘yes and they are important’,’ yes but they are moderate’, ‘yes but they are very moderate’,* and *‘no, I don't have any sequelae left’ (and no answer refusal, ‘not concerned’)*. Then, response categories were merged to form a three‐level variable (no sequelae, yes (very) moderate, and yes (very) important).

##### Health outcomes

Cancer‐related fatigue was evaluated using the fatigue subscale of the EORTC QLQ scale. This scale is an instrument for measuring quality of life specifically for cancer patients, which includes three items that assess fatigue during the past week. These three items resulted in a score from 0 to 100, higher scores indicating more severe fatigue. Studies have shown that a score of 40 or higher was indicative of clinically significant fatigue.^28^ Depression and anxiety were assessed using the HAD scale, an instrument for detecting anxiety and depressive disorders. It includes 14 items rated from 0 to 3. Seven questions relate to anxiety and seven others to the depressive dimension, allowing two scores to be obtained (maximum score of each score = 21). The HADS uses two threshold values, one for screening for "major" disorders (≥11) and the second for detecting "borderline" disorders (between 8 and 10).^29^ Pain is measured using pain felt during the last 15 days.

### 2.4 Statistical analyses

Mean and standard deviation (SD) were computed for continuous variables. Categorical variables were expressed as proportions. Univariate analyses were performed to compare respondents versus nonrespondents to the SILS and to compare participants depending on their HL level. ANOVAs were used to compare continuous variables while chi‐squared tests were conducted to compare categorical data. First, we looked at the relationships between HL and other determinants. Then to identify the factors associated with medico‐social follow‐up, binary logistic regression models were performed using a backward elimination procedure. All the variables found in the univariate analyses to be associated with a *P*‐value <.2 were tested in the multivariable model. All the statistical analyses were two tailed, and the results were taken to be statistically significant when *P*‐values <.05 were obtained. All the analyses were performed using IBM SPSS Statistics 18.0 (IBM Inc).

## 3 RESULTS

Of the 4 174 patients who participated in the VICAN5 study, 96.9% have completed the SILS. Nonrespondents to this question were more often men, with a professional activity. They also had lower educational attainment and lower income.

Patients’ characteristics are presented in Table 1. The mean age at diagnosis was 54.7 years (SD = 12.9), 62.7% were women, and 49.5% had a level of education greater than or equal to high‐school graduate. Breast cancer was the most frequent cancer site (41.6%) followed by prostate cancer (16.5%, Appendix A).

**TABLE 1:** Patient's sociodemographic characteristics by follow‐up by general practitioner and social worker (VICAN study, weighted data, N = 4045)

Sociodemographic characteristics	Overall population	General practitioner follow‐up		*P*	Contact with a social worker		*P*
	%	Yes (%)	No (%)		Yes (%)	No (%)	
Age at diagnosis							
Mean (SD)	54.7 (12.9)	55 (12.8)	54 (12.9)	.017	49.4 (11.7)	55.6 (12.9)	<.001
Gender							
Men	37.3	38.8	33.9		22.4	39.8	
Women	62.7	61.2	66.1	.003	77.6	60.2	<.001
Marital status at time of the survey							
Married/cohabitant/ civil partnership	71.2	69.7	74.1		56.6	73.7	
Single/divorced/separated/widow(er)	28.8	30.3	25.9	.004	43.4	26.3	<.001
Education level							
No diploma	5.9	6.6	4.5		7.7	5.7	
Lower than high‐school graduate	44.6	47.7	38.2		48.4	44.0	
Greater than or equal to high‐school graduate	49.5	45.7	57.3	<.001	43.9	50.4	.007
Health literacy at time of the survey							
Inadequate	20.0	22.2	15.7		28.7	18.4	
Marginal	17.0	18.7	13.4		16.4	17.1	
Adequate	63.0	59.1	70.9	<.001	54.9	64.5	<.001
Employment at time of the survey							
Employed	41.0	38.1	47.3		43.8	40.6	
Unemployment	4.2	5.1	2.4		9.1	3.3	
Retired	42.9	43.2	42.0		21.9	46.5	
Inactive	11.9	13.6	8.3	<.001	25.2	9.6	.001
Income at time of the survey							
Low^a^	22.3	24.6	17.5		42.6	18.9	
Medium	46.6	47.2	45.4		43.1	47.2	
High	23.7	21.6	28.2		9.6	26.1	
Unknown	7.4	6.6	8.9	<.001	4.8	7.8	<.001
Socioeconomic deprivation index of area of residence							
Low	25.6	24.7	27.4		23.2	26.0	
Medium	50.0	49.2	51.6		54.4	49.3	
High	24.4	26.1	21.0	.001	22.5	24.8	.075

^a^ Low: income < 1st quartile; Medium: 1st quartile ≤ income ≤ 3rd quartile; High: income > 3rd quartile.

Most cancer survivors (66.7%) were followed‐up by a GP after their initial cancer diagnosis. Concerning social worker follow‐up, only 14.5% had contact with a social worker since diagnosis. In the univariable analysis (Table 1), a lower level of HL increased significantly GP follow‐up and contact with social workers. Follow‐up by a GP was also more frequent for older men, with lower educational attainment, those living alone in socioeconomic deprived areas, and those with lower income and higher perceived precariousness (perceived financial comfort, Appendix B). Social workers were more often contacted by younger women, less often retired, with lower educational attainment, those living alone, and those with lower income and higher perceived precariousness. In addition, impaired health status (sequelae, fatigue, pain, depression, and anxiety) increased GP follow‐up and contact with social workers (Table 2).

**TABLE 2:** Patient's medical and psychosocial characteristics by follow‐up by general practitioner and social worker (VICAN study. weighted data. N = 4045)

Medical and psychosocial characteristics	Overall population	General practitioner follow‐up		*P*	Contact with a social worker		*P*
	%	Yes (%)	No (%)		Yes (%)	No (%)	
Cancer localization							
Breast	41.6	40.8	43.7		55.3	39.3	
Lung	3.4	4.3	1.6		4.5	3.2	
Colorectal	10.0	10.0	10.1		9.9	10.1	
Prostate	16.5	17.8	13.8		3.3	18.7	
Upper aero‐digestive tract	4.4	4.6	3.6		8.0	3.7	
Bladder	3.1	2.9	3.6		1.3	3.5	
Kidney	3.5	4.0	2.6		2.1	3.8	
Non‐Hodgkin lymphoma	3.8	3.5	4.5		4.7	3.7	
Melanoma	4.9	2.8	9.1		2.7	5.2	
Cervical	2.3	1.8	3.1		3.9	2.0	
Endometrial	1.2	0.9	1.8		1.0	1.2	
Thyroid	5.2	6.6	2.3	<.001	3.2	5.5	<.001
Initial treatment by chemotherapy							
No	61.4	60.3	63.3		34.5	65.9	
Yes	38.6	39.7	36.7	.069	65.5	34.1	<.001
Initial treatment by radiotherapy							
No	46.2	45.8	46.7		33.2	48.4	
Yes	53.8	54.2	53.3	.591	66.8	51.6	<.001
Metastasis at the time of diagnosis							
No	98.2	98.2	98		96.4	98.5	
Yes	1.8	1.8	2.0	.664	3.6	1.5	.002
Perceived sequelae at time of the survey							
No. no sequel	35.6	31.2	44.3		21.3	38.1	
Yes, moderate/ very moderate	41.2	42.1	39.4		38.2	41.7	
Yes, important/ very important	23.2	26.7	16.3	<.001	40.5	20.2	<.001
Fatigue (EORTC fatigue score ≥40) at time of the survey							
No	51.4	47.3	59.5		28.0	55.4	
Yes	48.5	52.7	40.5	<.001	72.0	44.6	<.001
Pain perceived in the last 15 d at time of the survey							
No	26.2	23.7	31.3		12.7	28.5	
Yes	73.8	76.3	68.7	<.001	87.3	71.5	<.001
Depression at time of the survey							
No depressive symptoms	83.0	80.2	88.6		70.4	85.1	
Borderline depression	10.5	12.4	6.6		15.7	9.6	
Major depression	6.5	7.4	4.8	<.001	13.8	5.3	<.001
Anxiety at time of the survey							
No anxiety symptoms	53.2	50.7	58.0		37.6	55.9	
Borderline anxiety	23.9	24.5	22.7		25.5	23.6	
Major anxiety	22.9	24.9	19.3	<.001	36.9	20.6	<.001

Overall, 37% of cancer survivors had a limited HL level: 20% had inadequate and 17% had marginal level. Limited HL ranged from 32.8% to 45.5% depending on cancer sites with higher prevalence among patients who had lung or upper aero‐digestive tract cancer (*P* = .014, Appendix A). The factors associated with HL are presented in Table 3. Respondents with a limited HL had a lower socioeconomic level. They were less educated, lived on lower incomes, and more often perceived their household's financial situation as difficult compared with people with an adequate HL level. However, no association was observed among HL, age, and the deprivation index of area of residence. In addition, limited HL was associated with several measures of perceived health. Respondents with limited HL reported more frequently sequelae, anxiety, depressive symptoms, pain, and fatigue.

**TABLE 3:** Factors associated with health literacy (HL) level (VICAN study, weighted data, N = 4040)

	Adequate HL (%)	Limited HL (%)	*P*
Sociodemographic characteristics			
Age at diagnosis			
Mean (SD)	54.9 (13.0)	54.6 (12.8)	.442
Education level			
No diploma	5.2	7.2	
Lower than high‐school graduate	42.9	47.3	
Greater than or equal to high‐school graduate	51.9	45.6	<.001
Income at time of the survey			
Low^a^	20.7	25.0	
Medium	46.2	47.4	
High	25.5	20.8	
Unknown	7.6	6.9	<.001
Perceived financial comfort			
Comfortable	16.2	12.2	
Okay	34.8	32.5	
Need to be careful	36.4	38.8	
Hard or debts	12.6	16.5	<.001
Socioeconomic deprivation index of area of residence			
Low	26.1	24.7	
Medium	50.2	49.7	
High	23.7	25.7	.320
Medical and psychosocial characteristics			
Anxiety at time of the survey			
No anxiety symptoms	56.7	47.3	
Borderline anxiety	22.7	25.9	
Major anxiety	20.6	26.8	<.001
Depression at time of the survey			
No depressive symptoms	85.9	78.1	
Borderline depression	8.2	14.2	
Major depression	5.8	7.7	<.001
Perceived sequelae at time of the survey			
No sequel	38.0	31.6	
Yes, moderate/ very moderate	40.6	42.1	
Yes, important/ very important	21.4	26.3	<.001
Fatigue (EORTC fatigue score ≥40) at time of the survey			
No	56.4	42.8	
Yes	43.6	57.2	<.001
Pain perceived in the last 15 d at time of the survey			
No	29.7	20.1	
Yes	70.3	79.9	<.001

^a^ Low: income < 1st quartile; Medium: 1st quartile ≤ income ≤ 3rd quartile; High: income > 3rd quartile.

Most of sociodemographic, medical, and psychosocial factors tested in the univariable analysis remained independently associated with medico‐social follow‐up in the multivariable analyses (Table 4). Regarding sociodemographic factors, follow‐up by a GP was more frequent for survivors with lower educational attainment. Social workers were more often contacted by younger people, those living alone and outside an area of high socioeconomic deprivation. However, several factors had the same impact on both medical and social follow‐up visits. Medico‐social follow‐up increased with a low income and unemployment. Despite it being associated with lower socio‐economic status and impaired health (Table 3), having low HL still increased the likelihood of the medico‐social follow‐up after multivariable adjustment.

**TABLE 4:** Multiple logistic regression analysis of follow‐up by general practitioner and contact with social worker

Survivors’ sociodemographic characteristics	Follow‐up by general practitioner (N = 3934)		Contact with social worker (N = 3948)	
	OR	95% CI	OR	95% CI
Age at diagnosis^a^	1.01	1.00‐1.02	0.98	0.96‐0.99
Marital status				
Married/cohabitant/ civil partnership			0.61	0.51‐0.78
Single/divorced/separated/widow(er)			1	
Education level				
No diploma	1.51	1.07‐2.12		
Lower than high‐school graduate	1.35	1.15‐1.59		
Greater than or equal to high‐school graduate	1			
Health literacy				
Inadequate	1.52	1.26‐1.84	1.40	1.11‐1.77
Marginal	1.58	1.30‐1.94	1.02	0.78‐1.35
Adequate	1		1	
Employment				
Employed	1.01	0.78 ‐ 1.31	0.99	0.67 ‐ 1.45
Unemployment	1.96	1.21 ‐ 3.16	1.66	1.00 ‐ 2.78
Retired	1		1	
Inactive	1.37	1.01 ‐ 1.88	1.62	1.10 ‐ 2.39
Income				
Low	1.34	1.07‐1.68	3.62	2.57‐5.11
Medium	1.16	0.97‐1.40	2.06	1.49‐2.83
High	1		1	
Unknown	0.84	0.63‐1.12	1.54	0.92‐2.59
Socioeconomic deprivation index				
Low			1	
Medium			1.08	0.85‐1.37
High			0.64	0.48‐0.85
Cancer localization				
Colorectal	1		1	
Breast	1.01	0.79‐1.30	1.11	0.78‐1.56
Lung	2.19	1.29‐3.73	0.93	0.53‐1.63
Prostate	1.62	1.20‐2.18	0.48	0.27‐0.86
Upper aero‐digestive tract	1.04	0.68‐1.59	1.2	0.72‐2.00
Bladder	0.84	0.54‐1.29	0.69	0.30‐1.58
Kidney	2.05	1.29‐3.25	0.97	0.48‐1.96
Thyroid	4.22	2.62‐6.78	0.52	0.28‐0.98
Non‐Hodgkin lymphoma	0.82	0.55‐1.23	0.96	0.55‐1.66
Melanoma	0.46	0.32‐0.68	0.83	0.44‐1.57
Cervical	0.58	0.36‐0.96	1.25	0.67‐2.33
Endometrial	0.56	0.30‐1.04	1.58	0.60‐4.19
Initial treatment by chemotherapy	1.24	1.05‐1.48	2.16	1.71‐2.73
Perceived sequelae				
No, no sequel	1		1	
Yes, but moderate/ very moderate	1.43	1.21‐1.68	1.14	0.88‐1.47
Yes, but important/ very important	1.84	1.49‐2.26	1.75	1.31‐2.32
Fatigue (EORTC fatigue score ≥40)	1.31	1.13‐1.53	1.54	1.22‐1.94
Pain felt in the last 15 d			1.33	1.10‐1.79
Depression				
No depressive symptoms	1		1	
Borderline depression	1.53	1.17‐2.00	1.35	1.00‐1.81
Major depression	0.98	0.71‐1.35	1.61	1.15‐2.25

^a^ Per 1‐point increase.

Furthermore, regarding medical factors, survivors with thyroid, lung, kidney, and prostate cancer were more likely to be followed by their GP while survivors with melanoma and cervical cancer were less followed. Prostate and thyroid cancer survivors were less likely to contact a social worker. Those who reported pain had more contact with a social worker only. In addition, treatment by chemotherapy, sequelae, depressive symptoms, and fatigue was associated with more frequent medico‐social follow‐up.

## 4 DISCUSSION

In this national representative study assessing the association between HL and medico‐social follow‐up among French cancer survivors 5 years postdiagnosis, our results show that cancer survivors have frequent limited HL level (37%), usual follow‐up visits to a general practitioner (66.7%), and scarce contact with a social worker (14.5%). Follow‐up visits with GPs were more frequent among cancer survivors with marginal or inadequate functional HL while social follow‐up was associated only with the highest difficulties to understand health‐related information (inadequate HL level).

Among the patients' concerns after treatment is the need to rebuild their lives. This obviously requires psychosocial support, the consideration of physical sequelae, and the elaboration of a new life project. This process must often also take into account the fatigue and sequelae that can linger for several years.^30^ On the other hand, some people will have to face the consequences of the loss of autonomy in terms of their physical or social capacities. Thus, long‐term follow‐up requires the collaboration of several professionals including GPs.^31^ This helps to reduce the burden of oncology services and to have a personalized follow‐up for each patient.

As expected, medical follow‐up by GP was associated with several indicators of cancer severity in multivariable analysis. Cancer survivors who experience sequelae and fatigue visited their GP, probably to help them overcome these difficulties.^13^ In addition, patients with suspected depressive state were followed‐up more frequently by a GP. Interestingly, this did not apply to those with more severe depressive symptoms, probably followed‐up directly by a psychiatrist or less likely to adhere to medical follow‐up as depressed patients may employ maladaptive coping strategies.^32^ Furthermore, thyroid, lung, kidney, and prostate cancer survivors were more often followed by their GP probably more often implicated in dealing with hormone replacement or side effects of treatment.^33^

It is true that follow‐up by a GP after treatment is appropriate if a survivorship care plan is well‐defined upstream.^5^ However, in a context where there is no model of survivorship care plan clearly defining the places and roles of GPs and specialists, a high rate of follow‐up by GP can be a marker inequality of access to cancer care ^34^ or a marker of large information needs among those who might have difficulties to understand their specialists.^35^

Our finding showed that after considering health status, patients with lower income and education and who were unemployed were more likely to consult their GP for their medical follow‐up. We hypothesize that the rising fees applied by French private medical specialists’ consultations could explain this finding and suggest unequal access to specialized care for cancer survivors with low income. This result is also consistent with those of previous studies that underlined the association between lower socioeconomic position and GP use.^12^, ^36^ However, additional data on the follow‐up of cancer survivors by specialists are needed in order to thoroughly document medical follow‐up in general.

Regarding HL, difficulties in accessing and understanding health information may not allow these patients to easily navigate the health‐care system.^19^, ^20^, ^21^, ^22^, ^23^ They might need to be informed or referred by their GPs. In addition, this result confirms previous study showing that low HL is associated with greater use of health‐care services including GP home consultation.^37^ For those patients, the GP is naturally their primary source of care and information. However, to date, little actions exist in France to ensure adequate medical care and effective communication to low‐literate patients.

Social support plays a leading role in adapting to and overcoming the difficulties associated with cancer survivorship.^38^ In our study, inadequate HL was associated with social follow‐up. Cancer survivors with inadequate HL can contact social workers for administrative matters related to the health‐care system and even to fill out medical forms. Indeed, their difficulties understanding and accessing information may require the assistance of a third person.^39^, ^40^ Social workers were also more often contacted by cancer survivors living alone. Some results of this study may indirectly raise question of financial difficulties and social needs of cancer survivors as observed in previous studies.^41^, ^42^ As expected, cancer survivors with lower income and unemployed contacted social workers to palliate financial difficulties or for support upon return to work. In France, the costs are generally borne by the national Health Insurance. Thus, financial difficulties are related mainly to the loss of employment, reduction of working time, or regular sick leave. However, cancer survivors living in an area with a high socioeconomic deprivation and older survivors reported fewer follow‐ups by social workers. These results might reflect the social exclusion of patients living in the deprived areas^43^ and the fact that older patients are less concerned by employment.

Furthermore, our results showed that cancer survivors with impaired health were followed more frequently by social workers. It suggests that a poor health status requires more help in adapting work, setting up the workstation or for home support for housework. The social workers can make the link between the patient and available services or programs that can facilitate his/her daily life, relieving him/her of tasks that illness or treatment prevents him/her from doing.^44^

Even after adjusting for several indicators of impaired health (and for multiple sociodemographic factors) associated with HL (Table 3), HL level remained significantly associated with medico‐social follow‐up. A previous study on patients with chronic illness has also showed that low functional HL was associated with high frequency of GP visit and this after adjusting for general health status.^45^ These results reinforce our assumption that patients with limited HL have medical needs related to their poor health status. In addition, they raise several questions about the information needs and understanding of this information for people with a limited level of HL.

Beside the strengths of this national representative study evaluating both medical and other characteristics in a large sample of cancer survivors, some limitations need to be underlined. We used a 1‐item subjective HL (SILS). Due to the length of the survey and an effort to minimize attrition by using short validated measures whenever available, it was not possible to use a longer measure of HL. However, it has been demonstrated to predict functional HL of different population including cancer survivors and general population in France.^46^, ^47^ In addition, the association between social variables might have caused multicollinearity. The exact frequency and motivations to consult a GP or a social worker were not collected. Indeed, the cross‐sectional nature of the questions analyzed and their wide timeframe do not allow to capture the density of GP and SW visits over time. Furthermore, some themes were not explored in the 5‐year survey, in particular the specific follow‐up by psychiatrists or psychologists. This raises questions about long‐term psychological care for cancer survivors. Future research should also include follow‐up by specialist physicians to assess equity in access to specialized care. Furthermore, a qualitative study exploring different reasons for cancer survivor consulting GP and contacting SW could help interpret some of our findings.

## 5 CONCLUSION

Among French cancer survivors, 37% had a limited HL level. Despite no specific survivorship care plans accounting for HL level, French cancer survivors with limited HL are more likely to consult their GP and contact a SW, such as survivors with lower socioeconomic status and more severe cancer. Future research will investigate if this type of follow‐up could help mitigate health inequities. The follow‐up of patients with inadequate HL requires specific attention to ensure effective communication and information exchange. Raising awareness and training GPs and SWs on medico‐social follow‐up for patients with limited HL seem necessary.

## AUTHOR CONTRIBUTIONS

Youssoufa M. Ousseine: Formal analysis, inestigation, methodology, software, visualization, and writing – original draft. Anne‐Déborah Bouhnik: Conceptualization, validation, methodology, and writing – review and editing. Patrick Peretti‐Watel: Conceptualization, project administration, writing – review and editing. Aline Sarradon‐Eck: writing – review and editing. Victoria Memoli: data management, writing – review and editing. Marc‐Karim Bendiane: Conceptualization, project administration, writing – review and editing. Marie‐Anne Durand: writing – review and editing. Julien Mancini: Conceptualization, validation, methodology, writing – original draft, review and editing.

## ETHICAL APPROVAL/INFORMED CONSENT

The study methodology was approved by three national ethics commissions: the CCTIRS (no 11‐143), the ISP (no C11‐63), and the CNIL (no 911290). Confidentiality is assured for all participants with regard to any personal responses and information provided, as all data collected are anonymized.

Informed consent was obtained from all individual participants included in the study.

## Data Availability

VICAN survey data are available through the French National Cancer Institute (INCa).
